# Aripiprazole treatment for temper outbursts in Prader–Willi syndrome

**DOI:** 10.1186/s13023-022-02470-y

**Published:** 2022-08-26

**Authors:** Maximilian Deest, Jelte Wieting, Maximilian Michael Jakob, Stephanie Deest-Gaubatz, Adrian Groh, Johanna Seifert, Sermin Toto, Stefan Bleich, Helge Frieling, Christian K. Eberlein

**Affiliations:** Department of Psychiatry, Social Psychiatry and Psychotherapy, Hannover Medical School, Carl-Neuberg-Straße 1, 30625 Hannover, Germany

**Keywords:** Prader–Willi syndrome, Aripiprazole, Mental health, Pharmacotherapy, Temper outbursts

## Abstract

**Background:**

Prader–Willi syndrome (PWS) is a rare neurodevelopmental disorder based on a loss of paternally expressed genes in chromosome segment 15q11-13. Behavioral traits such as temper outbursts, stereotypic, and ritualistic behavior, as well as an increased risk of psychosis accompany the syndrome, representing a major issue in the treatment of adults with PWS. Up to now, no treatment guideline for these conditions in PWS exist. This study aimed to retrospectively analyze the effect and adverse effects of treatment with aripiprazole for temper outbursts in 10 adults with PWS.

**Results:**

Aripiprazole was prescribed for temper outbursts (n = 10). Treatment outcome was assessed using the Clinical Global Impression-Severity (CGI-S) and -Improvement Scale (CGI-I).

Treatment success (CGI-I < 3) was observed in 70% of cases, with adverse effects from mild to partly serious extent in 60% of cases. The major adverse effect observed was increased daytime sleepiness. In total, 50% of the individuals were treated successfully for temper outbursts. The BMI did not change significantly in the successfully treated group after 6 months of treatment.

**Conclusions:**

Aripiprazole can be a treatment option for temper outbursts in people with PWS. Although a high rate of side effects was detected, their severity led to discontinuation in only 20% of the cases. Furthermore, the absence of weight gain makes aripiprazole interesting especially for the PWS population.

## Background

Prader–Willi syndrome (PWS) is a rare, genetically determined neurodevelopmental disorder with a certain behavioral phenotype and high frequency of psychiatric co-morbidities. PWS is caused by a lack of normally paternally expressed but maternally imprinted genes on chr15 q11-13. This can occur via three mechanisms: deletion of the paternal 15q11-13 region (delPWS, approximately 60% of cases), maternal uniparental disomy (mUPD, 35%), or by a rare imprinting center defect (IC) [1, 2]. Independent of genotype, newborns typically present with a failure to thrive, weak suck, and feeding difficulties. In later infancy, the feeding difficulties subside and give way to severe hyperphagia leading to life-threatening obesity if food intake is not controlled [3, 4]. PWS is also characterized by a hypothalamic-pituitary dysfunction with a growth hormone deficiency, central hypothyroidism, and hypogonadism [5, 6]. Nearly all individuals with PWS show a delay in motor and speech development as well as a mostly mild to moderate intellectual disability[7]. The behavioral phenotype of PWS is characterized by stubbornness, ritualistic and repetitive behavior, skin picking, and temper outbursts [8]. Furthermore, the prevalence of certain psychiatric comorbidities is not only higher than in the general population, but also higher than in other forms of intellectual disability [9, 10]. Individuals with PWS are especially at risk of developing psychosis, affective disorders and obsessive compulsive disorder (OCD) [11–13]. The prevalence of comorbid autism spectrum disease (ASD) [14] is also increased. It seems that a genotype-dependent pattern of distribution of mental disorders occurs with individuals of the mUPD subtype being at higher risk of psychosis and ASD [15].

Psychiatric comorbidities and behavioral problems negatively affect the quality of life of the affected individuals and their caregivers [16]. Concomitant behavioral problems correlate with higher perceived levels of stress in parents of children with PWS [17]. However, no official therapeutic guidelines or recommendations regarding the use of psychotropic medication exist. Psychotropic drugs, especially antipsychotic, antidepressant, and mood stabilizing drugs, are widely prescribed in the treatment of behavioral problems or mental disorders occurring in patients with PWS [9]. However, scientific literature on the efficacy and the adverse effects of psychotropic medication in PWS is sparse and mostly derives from case reports [18]. There are only a few case reports or case series regarding the use of antipsychotic drugs in PWS [19–21]. First-generation, as well as second-generation antipsychotic drugs, are used in PWS, neglecting their unfavorable pharmacological profile, such as high rates of extrapyramidal symptoms (EPS), weight gain, and hormone dysfunctions. The effect of newer antipsychotic drugs with a more favorable receptor profile such as aripiprazole is not well investigated. To our knowledge, only two case reports on treatment with aripiprazole in PWS currently exist. The first case presents an 11-year old girl with PWS with OCD and aggressive symptoms which subsided under treatment with 10 mg of aripiprazole per day [22]. The second case presents a 16-year-old female patient with PWS of the mUPD subtype who was treated with aripiprazole due to psychosis [23]. There are several rationales for considering aripiprazole as an effective treatment option in PWS. It is approved for the treatment of schizophrenia and bipolar disorder and used as an augmentation strategy in selective serotonin reuptake inhibitor (SSRI)-resistant OCD [24–27]. Furthermore, the range of indications include treatment of aggressive behavior in children with ASD and has shown promising effects in the treatment of psychosis in other rare genetic syndromes like 22q11.2 deletion syndrome [28, 29]. In comparison to other antipsychotic drugs, aripiprazole’s receptor affinity profile may be favorable as it is less likely to cause weight gain, EPS, or hyperprolactinemia.

### Aims of the study

In this study we retrospectively analyzed the treatment efficacy and adverse effects occurring under treatment with aripiprazole in 10 individuals with PWS. Treatment with aripiprazole was initiated for temper outbursts.

## Results

In this study, 10 individuals with PWS were treated with aripiprazole for temper outbursts. The age of participants ranged from 25 to 52 years with a mean age of 35.8 years (4 female, 6 male). In 6 cases PWS was caused by a delPWS. Although PWS was genetically confirmed in all cases, the genetic subtype (delPWS, mUPD, IC) was unknown in the remaining 4 cases. Out of the 10 participants, 7 were psychopharmalogical treatment naïve and 3 were currently treated with at least one other psychotropic drug (n = 1 antidepressant drug, and n = 2 antipsychotic drugs + antidepressant drugs).

### Baseline assessment

10 patients (6 males, 4 females) in this study sought evaluation and management for severe temper outbursts. Temper outbursts were characterized by irritability in general and tantrums during conflicts or disagreements. All the temper outbursts were accompanied by physical aggression with the destruction of property or attacking others.

At baseline, 2 participants showed a CGI of 5 (‘markedly ill’ = temper outbursts more than twice a month and/or severe damage delivered to things but not to living beings), 7 had a score of 6 (‘severely ill’ = multiple temper outburst per month and/or severe damage delivered to things or not serious injury of living beings) and 1 scored a 7 (‘among the most extremely ill patients' = multiple temper outburst per week and/or severe damage delivered to things or living beings).

### Treatment outcome

After treatment with aripiprazole, 3 patients showed a CGI-I of 1 (“very much improved), 4 showed a score of 2 (“much improved”), 2 showed an CGI-I of 3 (“minimal improved”), 1 scored 4 (“no change”). However, in 2 of the 7 patients who responded (CGI of 1 or 2), treatment with aripiprazole had to be discontinued because of adverse effects (see section adverse effects under treatment). Thus, 5 out of 10 (50%) patients were successfully treated with aripiprazole for temper outbursts. However, out of these 5 patients, n = 2 showed also adverse effects in form of increased daytime sleepiness. Out of the remaining 3 patients, 2 only improved minimally. ID 9 did not improve at all; unfortunately she refused to take a higher dose of aripiprazole, so the medication was discontinued.

Overall, 7 patients (70%) responded to aripiprazole showing a CGI-I of 1 or 2. Out of the 3 patients who already received treatment with at least one psychotropic drug at baseline, treatment with aripiprazole led to an improvement in all cases. It should be mentioned that ID 4 discontinued risperidone before receiving aripiprazole but sertraline was continued. ID 8 started sertraline 8 weeks after the beginning of treatment with aripiprazole due to depressed mood and anxiety. Among patients who responded to aripiprazole, 5 showed response with a dose of 5 mg per day and 2 with a daily dose of 10 mg. In 3 cases the treatment was discontinued, either because of adverse effects (n = 2) or because a sufficient improvement of symptoms could not be observed (n = 1).

### Observed adverse effects

All study participants underwent routine blood testing at the general practitioner and results were presented at the clinical visits. No abnormal electrolytes, blood cell counts, or other routine values were reported. Out of the 10 individuals treated with aripiprazole, 6 (60%) showed adverse effects among which increased daytime sleepiness was the most common (n = 6). In 2 out of these 6 patients, daytime sleepiness was only mild, so no change of medication was necessary. In 2 cases, switching administration to the evening reduced daytime sleepiness. In the remaining 2 cases, daytime sleepiness was so severe, that the medication with aripiprazole had to be discontinued although the patients had shown a positive response regarding temper outbursts. In 2 cases (20%) the daytime sleepiness was too severe even after changing the medication to the evening so that treatment had to be discontinued. No other adverse effects were observed during the time of this study. Especially the change of BMI was negligible after 6 months of treatment.

### BMI changes under treatment

Weight changes were analyzed in patients who underwent treatment for 6 months (n = 7) using the mean body mass index (BMI). Among these 7 patients, the BMI was 23.76 kg/m^2^ (min 21.11, max 26.35) at baseline and 23.49 kg/m^2^ (min 20.47, max 26.96) after 6 months of treatment. Thus, no remarkable change in BMI did take place under treatment.

## Discussion

Behavioral problems and mental disorders are frequent in PWS and are strongly associated with a reduced quality of life for individuals and their caregivers [9, 12, 16, 31, 32]. While they are considered the most challenging issue in adults with PWS [17], reliable treatment recommendations are currently not available and the scientific literature on the effectiveness and adverse drug reactions of psychotropic drugs in patients with PWS is sparse. Demographic studies have revealed that both first-generation as well as second-generation antipsychotic drugs are widely prescribed to patients with PWS [9]. Other commonly used psychotropic drugs include SSRIs, such as sertraline and fluoxetine, or antiepileptic drugs such as topiramate [33–36]. The reported treatment outcomes differ, showing a beneficial effect in some cases but also a worsening of symptoms in others [37]. Among antipsychotic drugs, there are only case reports and small case series showing promising effects of risperidone in the treatment of aggressive behavior [19, 20]. To our knowledge, there are only two case reports to date regarding the use of aripiprazole in patients with PWS. The first case report showed a therapeutic benefit in treating psychosis in a 16-year-old girl with PWS [23]. In the second case, an 11-year-old girl with OCD and aggressive behavior responded well to aripiprazole [22]. In our cohort of 10 patients with PWS, 70% of the patients responded to treatment with aripiprazole and showed a significant decrease of frequency and/or severity of temper outbursts. Although more than half of the study participants showed side effects (increased daytime sleepiness), they were either of mild intensity or were better tolerated after administering the medication in the evening. Due to side effects only 2 individuals discontinued the treatment with aripiprazole which leaves a total of 50% of the study participants which responded to and tolerated aripiprazole. One caveat when interpreting the findings in the responders is the additional treatment with sertraline in 2 individuals (ID 4 and ID 8). Especially for ID 4 a synergistic effect is conceivable. ID 8 showed a decrease of conflict and temper outburst before administering sertraline, but still a synergistic effect has to be considered on the long term. Due to the small number of participants, it is not possible to see a significant difference in improvement between the individuals with a combination of aripiprazole and sertraline compared to the ones with aripiprazole alone.

We recently showed that the SSRI sertraline is a promising treatment option for temper outbursts in PWS by significantly reducing the frequency and intensity of outbursts in around 92% of cases without causing any severe adverse drug reactions [38]. Comparing these results with the response rates of 50% under aripiprazole in this study, one may conclude that sertraline should be preferred over aripiprazole for the treatment of temper outbursts in PWS. On the one hand this is especially true when considering the high rate of adverse effects, on the other hand one has to acknowledge that 3 of the responding individuals had or have treatment with SSRIs or SNRIs. In this study, 6 (60%) patients experienced adverse effects ranging from mild to extended daytime sleepiness. It is known that these adverse drug reactions can occur under aripiprazole but their frequency and their long-lasting duration in our study population are irritating. Especially at the low dose of 5-10 mg aripiprazole used in this study. Forster et al. recently showed that genetic variations in the cytochrome P450 enzyme system frequently occur in PWS [39]. For instance, the cytochromes 2D6 and 3A4/5, both of which are involved in the metabolism of aripiprazole, are also affected. An impaired metabolism could contribute to the high number of adverse effects under treatment with aripiprazole in our study. Another explanation would be, that individuals with PWS are more susceptible to adverse effects because of neural disturbances as a cause of the neurodevelopmental disorder itself. It should also be considered that the concomitant use of other psychotropic drugs increase the risk of adverse effects. On the other hand, daytime sleepiness as the adverse effect reported is also one of the prominent symptoms in adults with PWS even without medication, which makes it hard to disentangle the contribution of aripiprazole to this symptom. However, daytime sleepiness is not a common adverse drug reaction in aripiprazole-treated patients not suffering from PWS.

A major concern when using psychotropic medication to treat patients with PWS is possible weight gain. In our study, no change of BMI during 6 months of treatment could be observed.

The underlying pathophysiological mechanisms of temper outbursts in PWS are not yet known, but disturbances of the serotonergic system are postulated [40, 41]. The fact that patients seem to respond better to SSRIs than to the anti-dopaminergic effects of antipsychotic drugs may further support the hypothesis of a dysregulated serotonin metabolism. However, it should be considered that aripiprazole also functions as a partial agonist at serotonergic receptors [42]. Thus, future studies are necessary to investigate the pathophysiological mechanisms of temper outbursts to identify more promising treatment options.

The limitations of our study consist of its open-label design, the lack of a control group, and its design as a case series. The low number of cases is also a limitation, but this is due to the rarity of the disease. However, the study currently includes the largest cohort of individuals with PWS undergoing aripiprazole treatment. Another limitation is the assessment of symptoms and improvement using the CGI, which is not PWS specific. This is caused by a lack of reliable psychometric assessments in German language for PWS.

## Conclusions

Aripiprazole can lead to a reduction of symptoms in individuals with PWS suffering from temper outbursts. However a high rate of side effects was reported, these led to discontinuation of aripiprazole in only 20% of the cases. Aripiprazole can be a treatment option for people with PWS suffering from temper outburst where SSRIs alone did not show good effects. This might lead to a change from SSRIs to aripiprazole or even the combination of SSRIs and aripiprazole, which is not an uncommon drug combination.

## Methods

### Study design

This study was conducted as a case series with thorough examination of the characteristics of study participants and treatment outcomes. All patients included in this study were treated in the Outpatient Department for Mental Health in Rare Genetic Syndromes of the Department of Psychiatry, Social Psychiatry, and Psychotherapy of Hannover Medical School between 2011 and 2019. All data obtained in this study were routinely assessed at the visits and were analyzed retrospectively.

### Participants

This study included 10 individuals with genetically confirmed PWS. All of them were treated at the Outpatient Department for Mental Health in Rare Genetic Disorders of the Department of Psychiatry, Social Psychiatry and Psychotherapy of Hannover Medical School, Hannover, Germany, between 2011 and 2019. The reasons for consultation in this study were maladaptive behaviors with temper outbursts. Counseling consisted of psychoeducational support for the families and caregivers as well as medical treatment. A special behavioral therapy was not offered during the time of the study.

### Assessment of symptoms

Data on demographic information, current symptoms, medical history, and medication history were gathered at baseline via a comprehensive interview with the patient and their caregiver(s) (e.g. parents). The Clinical Global Impression-Severity scale (CGI-S) was used to address the severity of symptoms. In the CGI-S, the clinician rates the patient relative to their experience. Rating of the CGI-S is performed on a 7-point scale ranging from 1 (normal) to 7 (among the most severely ill patients) [30]. In general, initiation of drug treatment was only considered when the patient exceeded a score of 4 (moderately ill) or higher at baseline. Operational definition of the CGI-S ratings was:

1: normal, not ill at all (= no temper outbursts); 2: borderline mentally ill (= high irritability but no temper outbursts); 3: mildly ill (= temper outburst from time to time without destruction of things or aggression towards living beings); 4: moderately ill (= temper outbursts from time to time including destructions of things or aggression towards living beings without severe damage done); 5: markedly ill (= temper outbursts more than twice a month and/or severe damage delivered to things but not to living beings); 6: severely ill (= multiple temper outburst per month and/or severe damage delivered to things or not serious injury of living beings); 7 among the most extremely ill patients (= multiple temper outburst per week and/or severe damage delivered to things or living beings).

Before treatment, the participants and their caregivers were informed about the drug and possible adverse drug reactions. If written consent was given, treatment with aripiprazole at a starting dose of 5 mg per day was initiated. Except for ID 4 and ID8 prior psychotropic medication was discontinued the day before the first dose of aripiprazole. Follow-up visitations to check for tolerability and efficacy were performed regularly (at least 4 weeks after initiation of medication or increase of dose). Treatment efficacy was assessed using the CGI-Improvement scale (CGI-I) based on the information given by the patient and the caregivers during consultation [30]. The CGI-I addresses the global improvement compared to the subject’s condition at baseline using a 7-point scale ranging from 1 to 7. (compared to the patient's condition at admission, it very much improved since the initiation of treatment (CGI-I = 1); much improved (CGI-I = 2); minimally improved ((CGI-I = 3); no change from baseline(CGI-I = 4);; minimally worse (CGI-I = 5); much worse ((CGI-I = 6); very much worse since the initiation of treatment (CGI-I = 7. The CGI-I was assessed during all consultations. The endpoint of this study was defined as 6 months after treatment or prior to stopping medication. Patients were considered responsive to treatment when a CGI-I score of 1 or 2 was achieved. Assessment of adverse drug reactions was performed by interviewing the caregivers and the patient (Table 1).

**Table 1:** Characteristics of study participants and treatment outcome.

ID	Sex	Age	Genetic subtype	Symptoms baseline	CGI	Course	CGI-I	Dose	Side effects	BMI baseline kg/m^2^	BMI after 6 month kg/m^2^	Psychiatric medication	Notes
1	f	28	Unknown	Perseveration, obsessions. Treated with risperidone but severe daytime sleepiness. After stop of risperidone: massive temper outbursts	7	Decrease of intensity and frequency of outbursts	2	5 mg	Daytime sleepiness but less than with risperidone	22.19	22.60		Medication changed to dose in the evening to improve daytime sleepiness
2	m	31	delPWS 2	Irritability, temper outbursts	6	Irritability improved, nearly no more temper outbursts	1	5 mg	Daytime sleepiness	21.11	21.66		Medication changed to dose in the evening to improve daytime sleepiness
3	m	30	delPWS 1	Irritability, temper outbursts	6	Slight reduction in frequency and intensity of outbursts	3	5 mg	Mild increase of daytime sleepiness	26.12	24.82		
4	m	30	unknown	Irritability with severe temper outbursts, perseverations, obsessions, mistrust,	6	Improvement of outbursts, obsessions, mistrust	1	10 mg		26.34	26.96	risperidone 4 mg sertraline 100 mg (stopped at start of aripiprazole)	After 1 month significant improvement, then deterioration. After increase of dose again significant improvement
5	f	35	unknown	Irritability with temper outbursts	6	Improvement of irritability and temper outbursts	2	10 mg		21.78	20.47	milnacipram 50 mg (stopped at start of aripiprazole)	
6	m	52	delPWS 1	Irritability with temper outbursts with destruction of property. Conflicts with people	5	Improvement of irritability and temper outbursts	3	10 mg	Mild increase of daytime sleepiness	26.35	25.33		
7	f	46	delPWS 2	Irritability with temper outbursts	6	Improvement of irritability and outbursts	2	5 mg	Daytime sleepiness	25.11	/		Stopped because of side effects
8	m	25	delPWS	Irritability, temper outbursts with physical attacks	6	No conflicts anymore	1	5 mg		22.43	22.57	sertraline 50 mg risperidone 0.5 mg	Under Sertraline and Risperidone no treatment success, change of Risperidone to Aripiprazole lead to a massive improvement
9	f	37	Unknown	Irritability with temper outbursts	6	No change	4	5 mg		39.48	/		Stopped because no success
10	m	44	delPWS 2	Irritability with temper outbursts	5	Decrease of frequency & intensity of outbursts	2	5 mg	Daytime sleepiness	24.91	/		Stopped because of side effects

*CGI* clinical global impression scale; *CGI-I* clinical global improvement scale; *delPWS* paternal deletion

## Data Availability

The datasets generated and/or analysed during the current study are not publicly available due to data privacy reasons but are available from the corresponding author on reasonable request.

## References

[1] Butler MG, Miller JL, Forster JL (2019). Prader–Willi syndrome - clinical genetics, diagnosis and treatment approaches: an update. Curr Pediatr Rev.

[2] Horsthemke B, Wagstaff J (2008). Mechanisms of imprinting of the Prader–Willi/Angelman region. Am J Med Genet Part A.

[3] Miller JL, van Wattum PJ, Brokamp E, Fairbrother L, Scheimann A, Shoemaker AH (2018). A multidisciplinary approach to the clinical management of Prader–Willi syndrome. Mol Genet Genomic Med.

[4] Cassidy SB, Schwartz S, Miller JL, Driscoll DJ (2012). Prader–Willi syndrome. Genet Med.

[5] Tauber M, Coupaye M, Diene G, Molinas C, Valette M, Beauloye V (2019). Prader–Willi syndrome: a model for understanding the ghrelin system. J Neuroendocrinol.

[6] Angulo MA, Butler MG, Cataletto ME (2015). Prader–Willi syndrome: a review of clinical, genetic, and endocrine findings. J Endocrinol Invest.

[7] Dykens E, Shah B (2003). Psychiatric Disorders in Prader–Willi Syndrome. CNS Drugs.

[8] Steinhausen HC, Eiholzer U, Hauffa BP, Malin Z (2004). Behavioural and emotional disturbances in people with Prader–Willi syndrome. J Intellect Disabil Res.

[9] Soni S, Whittington J, Holland AJ, Webb T, Maina E, Boer H (2007). The course and outcome of psychiatric illness in people with Prader–Willi syndrome: Implications for management and treatment. J Intellect Disabil Res.

[10] Holland AJ, Whittington JE, Butler J, Webb T, Boer H, Clarke D (2003). Behavioral phenotypes associated with specific genetic disorders: evidence from a population-based study of people with Prader–Willi syndrome. Psychol Med.

[11] Soni S, Whittington J, Holland AJ, Webb T, Maina EN, Boer H (2008). The phenomenology and diagnosis of psychiatric illness in people with Prader–Willi syndrome. Psychol Med.

[12] Feighan SM, Hughes M, Maunder K, Roche E, Gallagher L (2020). A profile of mental health and behaviour in Prader–Willi syndrome. J Intellect Disabil Res.

[13] Manzardo AM, Weisensel N, Ayala S, Hossain W, Butler MG (2018). Prader–Willi syndrome genetic subtypes and clinical neuropsychiatric diagnoses in residential care adults. Clin Genet.

[14] Dykens EM, Roof E, Hunt-Hawkins H, Dankner N, Lee EB, Shivers CM (2017). Diagnoses and characteristics of autism spectrum disorders in children with Prader–Willi syndrome. J Neurodev Disord.

[15] Vogels A, Matthijs G, Legius E, Devriendt K, Fryns JP (2003). Chromosome 15 maternal uniparental disomy and psychosis in Prader–Willi syndrome [7]. J Med Genet.

[16] Mazaheri MM, Rae-Seebach RD, Preston HE, Schmidt M, Kountz-Edwards S, Field N (2013). The impact of Prader–Willi syndrome on the family’s quality of life and caregiving, and the unaffected siblings’ psychosocial adjustment. J Intellect Disabil Res.

[17] Lanfranchi S, Vianello R (2012). Stress, locus of control, and family cohesion and adaptability in parents of children with down, williams, fragile x, and Prader–Willi syndromes. Am J Intellect Dev Disabil.

[18] Bonnot O, Cohen D, Thuilleaux D, Consoli A, Cabal S, Tauber M (2015). Psychotropic treatments in Prader–Willi syndrome: a critical review of published literature. Eur J Pediatr.

[19] Durst R, Rubin-Jabotinsky K, Raskin S, Katz G, Zislin J (2000). Risperidone in treating behavioural disturbances of Prader–Willi syndrome. Acta Psychiatr Scand.

[20] Araki S, Ohji T, Shiota N, Dobashi K, Shimono M, Shirahata A (2010). Successful risperidone treatment for behavioral disturbances in Prader–Willi syndrome. Pediatr Int.

[21] Clarke D, Boer H, Webb T, Scott P, Frazer S, Vogels A (1998). Prader–Willi syndrome and psychotic symptoms: I. Case descriptions and genetic studies. J Intellect Disabil Res.

[22] Akça ÖF, Yilmaz S (2016). Aripiprazole in the treatment of obsessive compulsive disorder and aggressive behaviors in a child with prader willi syndrome. J Clin Psychopharmacol.

[23] Briegel W (2018). Clinical usefulness of aripiprazole treatment in a girl with Prader–Willi syndrome and psychosis. Clin Psychopharmacol Neurosci.

[24] Thamby A, Jaisoorya TS (2019). Antipsychotic augmentation in the treatment of obsessive-compulsive disorder. Indian J Psychiatry.

[25] Brakoulias V, Starcevic V, Albert U, Arumugham SS, Bailey BE, Belloch A (2019). Treatments used for obsessive–compulsive disorder—An international perspective. Hum Psychopharmacol.

[26] Di Sciascio G, Riva MA (2015). Aripiprazole: from pharmacological profile to clinical use. Neuropsychiatr Dis Treat.

[27] Shapiro DA, Renock S, Arrington E, Chiodo LA, Liu LX, Sibley DR (2003). Aripiprazole, a novel atypical antipsychotic drug with a unique and robust pharmacology. Neuropsychopharmacology.

[28] Rizzo R, Pavone P (2016). Aripiprazole for the treatment of irritability and aggression in children and adolescents affected by autism spectrum disorders. Expert Rev Neurother.

[29] Kontoangelos K, Lazaratou E, Economou M, Yiannopoulou KG, Papageorgiou CC (2020). Efficacy of low-dose aripiprazole for treatment of psychotic symptoms in a patient with 22q11.2 deletion syndrome. Psychopharmacol Bull.

[30] Guy W (1976). Clinical global impression (CGI). ECDEU Assess Man Psychopharmacol.

[31] Dimitropoulos A (2010). Clinical management of behavioral characteristics of Prader&ndash; Willi syndrome. Neuropsychiatr Dis Treat.

[32] Wieting J, Eberlein C, Bleich S, Frieling H, Deest M (2021). Behavioural change in Prader–Willi syndrome during COVID‐19 pandemic. J Intellect Disabil Res.

[33] Dech B, Budow L (1991). The use of fluoxetine in an adolescent with Prader–Willi Syndrome. J Am Acad Child Adolesc Psychiatry.

[34] Benjamin E, Buot-Smith T (1993). Naltrexone and fluoxetine in Prader–Willi Syndrome. J Am Acad Child Adolesc Psychiatry.

[35] Shapira NA, Lessig MC, Lewis MH, Goodman WK, Driscoll DJ (2004). Effects of topiramate in adults with Prader–Willi syndrome. Am J Ment Retard.

[36] Smathers SA, Wilson JG, Nigro MA (2003). Topiramate effectiveness in Prader–Willi syndrome. Pediatr Neurol.

[37] Kohn Y, Weizman A, Apter A (2001). Aggravation of food-related behavior in an adolescent with Prader–Willi Syndrome treated with fluvoxamine and fluoxetine. Int J Eat Disord.

[38] Deest M, Jakob MM, Seifert J, Bleich S, Frieling H, Eberlein C (2020). Sertraline as a treatment option for temper outbursts in Prader–Willi syndrome. Am J Med Genet Part A.

[39] Forster J, Duis J, Butler MG (2021). Pharmacogenetic testing of cytochrome P450 drug metabolizing enzymes in a case series of patients with Prader–Willi syndrome. Genes (Basel).

[40] Davies JR, Wilkinson LS, Isles AR, Humby T (2019). Prader–Willi syndrome imprinting centre deletion mice have impaired baseline and 5-HT2CR-mediated response inhibition. Hum Mol Genet.

[41] Kishore S, Stamm S (2006). Regulation of alternative splicing by snoRNAs. Cold Spring Harb Symp Quant Biol.

[42] Kikuchi T, Tottori K, Uwahodo Y, Hirose T, Miwa T, Oshiro Y, Morita S (1995). (OPC-1 4597), a new putative antipsychotic drug with both presynaptic dopamine autoreceptor agonistic activity and postsynaptic D2 receptor antagonistic activity. J Pharmacol Exp Ther.

